# Combined analysis of lncRNAs and mRNAs associated with coloration and wax formation during ‘Fumei’ Apple development

**DOI:** 10.1186/s12870-025-06545-3

**Published:** 2025-04-21

**Authors:** Wenping Huo, Shasha Liu, Xiao Chen, Tingting Gu, Zhongkang Wang, Xiaolong Xu, Daliang Liu, Yugang Zhang, Shenghui Jiang

**Affiliations:** 1 College of Horticulture, Engineering Laboratory of Genetic Improvement of Horticultural Crops of Shandong Province, Qingdao Agricultural University, Qingdao, Shandong China; 2 College of Agronomy, Qingdao Agricultural University, Qingdao, Shandong China; 3 College of Agricultural Sciences and Technology, Shandong Agriculture And Engineering University, Jinan, Shandong China; 4 Yantai Academy of Agricultural Sciences in Shandong Province, Yantai, Shandong China

**Keywords:** Apple, Anthocyanin, Wax, lncRNAs

## Abstract

‘Fumei’ apple is characterized by high anthocyanin content and thick wax layer. Long non-coding RNAs (lncRNAs) play essential roles in the growth and development of various plants via regulation of gene expression. This study explored the potential mechanism underlying anthocyanin accumulation and cuticular wax formation during the development of ‘Fumei’ apple fruit. The results demonstrated that anthocyanin accumulation correlates with fruit coloration, while wax content drives wax layer formation. A total of 6039 and 3410 differentially expressed genes (DEGs), as well as 230 and 131 differentially expressed lncRNAs (DELs) were identified in the M1/M2 and M2/M3 pairs, respectively, by using RNA-seq. In the M1/M2 pair, the DEGs were mainly enriched in the ‘photosynthesis’ and ‘flavonoid biosynthesis’ pathways; in the M2/M3 pair, the DEGs were significantly enriched in the ‘photosynthesis’ and ‘cutin, suberine and wax biosynthesis’ pathways. Furthermore, the structural and regulatory genes involved in anthocyanin and cuticular wax biosynthesis were investigated, and the potential lncRNAs and genes that may control the anthocyanin and cuticular wax biosynthesis were identified. This study provides candidate lncRNAs and potential regulatory genes associated with both the regulation of anthocyanins and wax during apple development.

**Supplementary Information:**

The online version contains supplementary material available at 10.1186/s12870-025-06545-3.

## Background

Apple is one of the most widely cultivated fruits in the world, particularly in China. The cultivation area and yield of apple in China exceed more than 50% of the global area and yield. Fruit quality is an important economic trait of apple, which directly affects the market value of apple. The fruits of most apple varieties are red, smooth and waxy as those traits are attractive to consumers [1, 2]. Additionally, red apples are rich in anthocyanins, the major compounds in the flavonoid pathway, while waxy apples are covered with cuticular wax [3–5].

It has been demonstrated that the content of anthocyanins determines the red pigmentation of apples. Anthocyanins are biosynthesized on the cytosolic surface of the endoplasmic reticulum (ER) by anthocyanin biosynthetic enzymes, which are encoded by structural genes [6]. They are transported into the vacuoles by the anthocyanin transporter proteins [7–9]. These structural genes can be divided into early biosynthetic genes (EBGs, such as *CHS*, *CHI*, and *F3H*) and late biosynthetic genes (LBGs, such as *DFR*, *ANS*, and *UFGT*) [6, 10]. To date, three anthocyanin transporters (ATP-binding cassette (ABC), glutathione S-transferase (GST), and multidrug and toxic compound extrusion (MATE)) have been identified in plants [9, 11, 12]. Anthocyanin biosynthesis is mainly controlled by regulatory genes, including MYBs, bHLHs and WD40 proteins, via generation of the MBW ternary complexes [13]. For instance, MdMYB1 is a key regulator in apple as it dominates anthocyanin biosynthesis on the fruit skin and interacts with MdbHLH3/33 [14, 15]. On the other hand, some negative regulators have been identified in apple (such as MdMYB6 and MdMYB16), strawberry (such as FvMYB1) and ginkgo (such as GbMYBF2) [16–19]. Furthermore, other transcription factors (TFs) can directly or indirectly regulate anthocyanin biosynthesis. For instance, PpBBX16 regulates light-induced anthocyanin biosynthesis by binding to the *PpMYB10* promoter in pear [20]; and MdEIL1 promotes anthocyanin biosynthesis by binding to the *MdMYB1* promoter in apple [21].

Covering the fruit surface, cuticular wax can effectively reduce post-harvest water loss and prevent pathogen invasion, thereby improving the appearance of fruits. In plants, cuticular wax biosynthesis generally comprises three steps: (1) *de novo* synthesis of C16 and C18 fatty acids [22]; (2) extension of very long chain fat acids (VLCFAs) [23, 24]; (3) formation of VLCFA derivatives, including alkanes, aldehydes, alcohols, ketones, and esters [25]. Different enzyme-coding genes have been isolated for each step. For instance, fatty acyl-ACP thioesterases (FATA and FATB) trigger the hydrolysis of C16–C18 acyl-acyl carrier proteins (ACP) into C16–C18 fatty acids in plastids, and the products are then activated by long-chain acyl-CoA synthetases (LACS) and exported to the ER [26]. The fatty acid elongase (FAE) complex is responsible for catalyzing the sequential reactions, including dehydration, reduction, condensation and second reduction, wherein KCS, KCR, ECR and HCD are the functioning enzymes [23, 24]. Finally, the wax components synthesized on the ER are transported to the plasma membrane (PM) by the ABC transporters and LTPGs [27, 28]. The wax biosynthesis is essentially regulated by transcriptional regulation in plants. For instance, the AP2-type TF WIN1/SHN1 promotes cuticular wax accumulation by regulating *KCS1*, *CER1* and *CER2* in *Arabidopsis* [29, 30]. Three homologous genes (AtMYB96, MdMYB94 and CsMYB96) are involved in drought stress response and wax biosynthesis [31–33]. In apple, the MYB TF MdMYB106 can improve the wax content by interacting with the *MdCER2* and *MdCER2L1* promoters and undergo MdBT2-mediated degradation under nitrogen signals [34].

The molecular mechanisms underlying anthocyanin accumulation and cuticular wax biosynthesis have been extensively studied in various plant species. However, the interplay between these two pathways remains largely unexplored. In this study, coloration and cuticular wax biosynthesis of apples were investigated based on the ‘Fumei’ apple. Specifically, transcriptome analysis was employed to identify DEGs and lncRNAs at different developmental stages of ‘Fumei’ apple. Functional analysis revealed that the DEGs were mainly enriched in the ‘Flavonoid’ and ‘Cutin, suberin and wax biosynthesis’ pathways. The *cis*-regulated target genes of DELs were significantly enriched in the ‘response to UV-B’ pathway and other stress-related pathways. Also, MdMYB94 was found to participate in both the biosynthesis of anthocyanins and wax. This study provides candidate genes and lncRNAs for the improvement on coloration and wax formation of apple fruits.

## Materials and methods

### Materials

‘Fumei’ apple fruit were obtained from a local orchard, which is a breeding base of Qingdao Agricultural University in Qingdao, China. A total of twenty ‘Fumei’ apple trees with a row spacing of 1.2 m × 3.5 m (4-year-old) were selected for fruit collection. The fruit were bagged on the 25th May 2022 (at 30 days after full bloom; DAFB) and the bags were removed at 160 DAFB. The fruits were collected at five stages including 0 days after bags removal (DABR), 6 DABR, 12 DABR, 18 DABR and 24 DABR. At each time 20 fruits of uniform size without mechanical damages were sampled. The peels were collected and stored at − 80 °C.

### Measurement of total content of anthocyanins

The content of anthocyanins was measured by a method reported previously [35]. First, 0.5 g of the fruit peel was collected and ground into a fine powder, followed by extraction in 10 mL methanol with 1% HCl for 24 h. Then, 1 mL of the extract was added into 4 mL of KCl buffer (0.025 M; pH = 1.0) and 4 mL of NaAc buffer (0.4 M; pH = 4.5), respectively. The two solutions were mixed and kept untouched at 4 °C for 15 min in the dark. Finally, the absorbance of the solution was measured at 510 and 700 nm, respectively. The content of anthocyanin was calculated by OD = (A_510_– A_700_) − 0.1 × (A_510_– A_700_).

### Characterization by scanning electron microscopy

The fruit skin was cut into a 0.5 × 0.5 cm pieces and fixed in the FAA solution. Then, the pieces were dehydrated using acetone. After the critical-point dried and gold spraying, the samples were checked by SEM (JSM-7500 F).

### Extraction and GC-MS analysis of cuticular wax

The fruit samples were immersed in chloroform to extract the cuticular wax. Then, the solution was concentrated using a rotary evaporator at 40 °C. After drying under N_2_ flow, the waxes were isolated and stored at − 80 °C until use.

The wax samples were dissolved in the chloroform and methanol solution (v: v = 9: 1) and mixed with 1 mg of n-tetracosane (Sigma Aldrich, China) as internal standard. After drying under N_2_ flow, the samples were mixed with bis-N, O-(trimethylsilyl) trifluoroacetamide (BSTFA, Sigma Aldrich, China) at 70 °C for 40 min. After that, the samples were injected into the GC-MS (QP-2020, Shimadzu, Tokyo, Japan) for further analysis. The NIST 17 MS Library was employed to identify the detected compounds. Additionally, the content of cuticular wax was calculated by using the internal standard method.

### RNA isolation, library construction, and sequencing

RNAprep Pure Plant Kit (Tiangen, Beijing, China) was used to isolate total RNA of the samples. Firstly, DNA and rRNA were removed from the total RNA samples. Then, the RNA was broken into 300–350 bp fragments and used as templates for synthesis of the first-strand cDNA by the random primer method. After removing the RNAs using the RNase H, the second-strand cDNA was synthesized. The double stranded cDNA was purified and end-repaired, followed by addition of the adapters. Finally, PCR was employed to construct a specific library. The Illumina NovaSeq6000 platform was employed for sequencing.

### Read mapping and transcriptome assembly

The raw data were generated by the sequencing, and adapters and low-quality reads were eliminated to obtain clean reads. The clean data were aligned to the GDDH13 apple genome [36] using HISAT2 software [37]. The StringTie software was employed to reconstruct the single sample transcript, while the Fragments Per Kilobase of transcript per Million fragments mapped (FPKM) was calculated by using the StringTie [38]. Additionally, the StringTie-Merge tool was used to combine transcripts from different samples. The raw sequencing data are publicly accessible under GSA No. CRA016451 (https://ngdc.cncb.ac.cn/gsa/browse/CRA016451) at https://ngdc.cncb.ac.cn/gsa.

### Identification of LncRNAs

The FEELnc tool was used to identify lncRNAs, and the protein-encoding capability of the lncRNAs was predicted by using computational tools including CPC2, PLEK, CNCI, Pfam and HMMER. The lncRNAs candidates were screened based on the following criteria: transcript length ≥ 200 bp, with two or more exons, and FPKM ≥ 0.1.

### Differential expression analysis of LncRNAs and encoding genes

The expression of lncRNAs and encoding genes was analyzed by the DESeq2 *R* package [39]. DEGs were analyzed with the following parameters:|log_2_FC| ≥ 1 and *p* value ≤ 0.05. Then, the DEGs were investigated by Gene Ontology (GO) and Kyoto Encyclopedia of Genes and Genomes (KEGG) analysis with the enrichGO and clusterProfiler packages, respectively.

### Prediction of target genes for LncRNAs

The predicted target genes were divided into *cis-* and *trans*-regulated genes. Herein, genes within 100 Kb upstream and downstream of lncRNAs were taken as potential *cis-*regulated target genes, while the genes whose expression is correlated with the lncRNAs were regarded as *trans*-regulated target genes.

### RT-PCR analysis

Total RNA of the apple peels was extracted by using the RNAprep pure Plant Kit (Tiangen, Beijing, China) and cDNA was synthesized by using the PrimeScript™ 1st strand cDNA Synthesis Kit (Takara, Beijing). All primers were provided by Shanghai Sangon Biotech Co. in China (Table S1). RT-PCR was performed with three biological and three technical replicates for each sample, and the results were analyzed by using the CFX96 system (BioRad, Hercules, CA, USA). The program was set as follows: 95 °C for 30 s, followed by 38 cycles of 95 °C for 10 s, and finally 58 °C for 30 s. *MdACTIN* was used as internal controls for gene expression. The transcription levels of genes were determined by a method reported previously [40].

### Statistical analysis

Data are presented as mean ± standard deviation (SD) from three independent experiments. Significant differences between groups were determined by one-way ANOVA followed by Tukey’s post-hoc test (*p* < 0.05) using the SPSS 22 software (IBM Corp., Armonk, NY, USA).

## Results

### Content of anthocyanins during fruit coloration

**Fig. 1:**
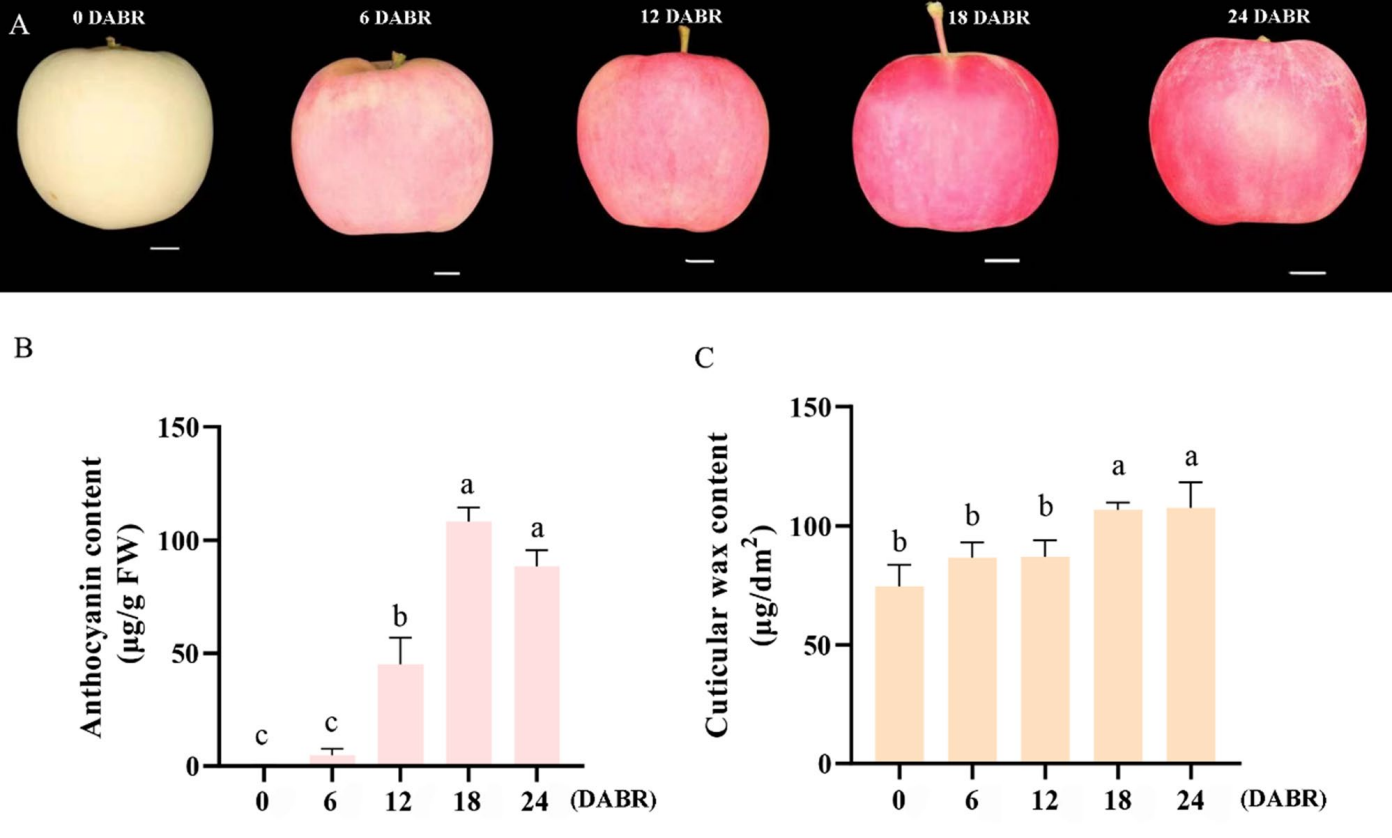
Development pattern of ‘Fumei’ apple fruit. **A**, Fruit phenotypes of ‘Fumei’ at different development stages. **B**, Anthocyanin contents in fruit peels of ‘Fumei’. **C**, Total wax contents in fruit peels of ‘Fumei’. Error bar means ± SD, *n* ≥ 3 and different letters indicate significant differences as assessed by one-way ANOVA (with Tukey’s test) (*p* < 0.01)

‘Fumei’ apple was selected as the study subject due to its characteristic red appearance and thick wax layer when harvested late (Fig. S1). In this study, we collected ‘Fumei’ fruit samples at five developmental stages (0, 6, 12, 18, 24 DABR). The results showed that after bag removal, the fruit color gradually turned red and the fruit appearance became shiny (Fig. 1A). Then, we measured the contents of anthocyanins in the fruit at the five developmental stages. The results showed that the content of anthocyanins gradually increased steadily until 18 DABR and decreased with time from 24 DABR (Fig. 1b).

### Changes in cuticular wax structure of ‘fumei’ fruit

**Fig. 2:**
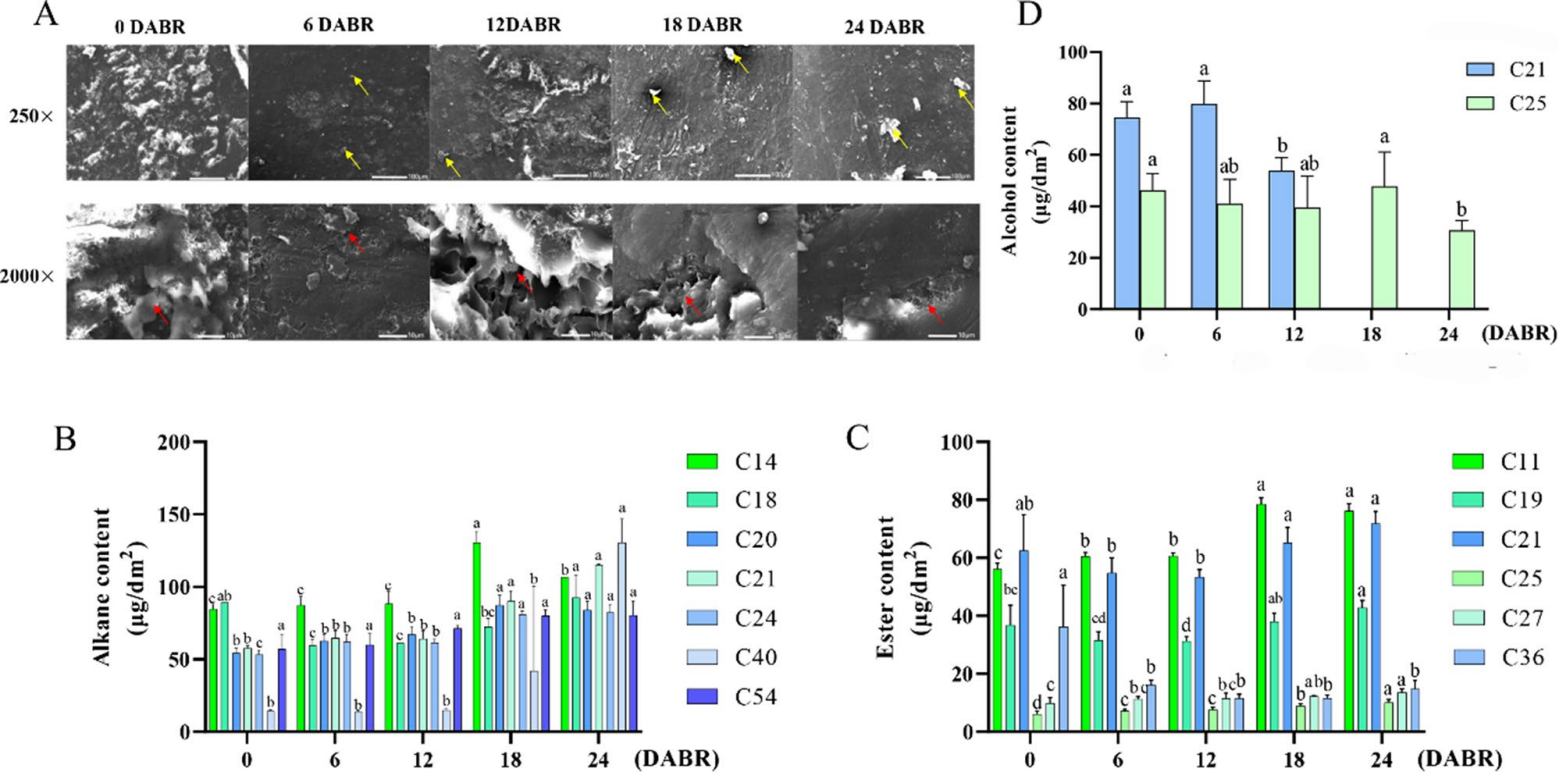
SEM images and the wax components of fruit peels of ‘Fumei’. **A**, SEM images of fruit peels of ‘Fumei’. The contents of alkane (**B**), alcohol (**C**) and wax ester (**D**) in fruit peels of ‘Fumei’. Error bar means ± SD, *n* ≥ 3 and different letters indicate significant differences as assessed by one-way ANOVA (with Tukey’s test) (*p* < 0.01)

The cuticular wax structure on ‘Fumei’ apple was investigated after bag removal. The SEM images (250×) showed deposit of cuticular wax on the fruit surface after bag removal. Also, crystals and small plates were observed at 6 DABR and the plates grew larger (Fig. 2A). Moreover, we also checked the structure of cuticular wax by high-power microscopy field (2500×). The wax layer on the fruit surface grew larger and thicker with time, especially at 6 and 12 DABR, but no wax films were generated at this stage. From 18 DABR, wax films were generated, which were stacked into layers covering the fruit surface (Fig. 2A).

### Cuticular wax content and composition on ‘fumei’ fruit

The total cuticular wax content was measured. The results indicated that the content of cuticular wax increased from 18 DABR and reached the peak at 24 DABR (Fig. 1C). Interestingly, the total content of cuticular wax was high at the beginning. Furthermore, the composition of cuticular wax on ‘Fumei’ fruit was determined by gas chromatography-mass spectrometry (GC-MS). The results showed that alkane was the predominant wax component in ‘Fumei’ fruit. Specifically, the content of C14 alkane was the highest at 18 DABR; the contents of C21 and C24 alkane increased at the last two stages (Fig. 2B); and those of C11, C19 and C21 wax ester increased drastically at the final two stages (Fig. 2C). However, the alcohol content decreased with over time during the development of ‘Fumei’ fruit (Fig. 2D). Overall, the increased content of alkane contributed to the accumulation of cuticular wax in ‘Fumei’ fruit.

### RNA-seq analysis of different developmental stages of ‘fumei’ fruit

To explore the DEGs of the anthocyanin and cuticular wax pathways in ‘Fumei’ fruit peels, the samples were performed by RNA sequencing at different developmental stages. Herein, the content of anthocyanins in the fruit increased drastically at 12 DAFR, and that of cuticular wax was maximized at 24 DAFR (Fig. 1A). Thus, 3 stages (0, 12,24 DAFR marked as M1, M2, M3) with three replicates for each stage were used for RNA-Seq analysis. Transcript abundance with at least two-fold changes and *p* < 0.05 denoted DEGs. A total of 6039 (2857 upregulated and 1389 downregulated) and 3410 (1389 upregulated and 2021 downregulated) DEGs were identified in the M1/M2 and M2/M3 pairs, respectively (Fig. S2 A, B, C).

GO analysis classified DEGs into three major categories: biological process, cellular component and molecular function, respectively. The results indicated that most genes related to the biological process were involved in the ‘response to chitin’, ‘photosynthesis’ and ‘photosynthesis, light reaction’ pathways. Many genes corresponding to the cellular component were related to the ‘plastid thylakoid membrane’, ‘chloroplast thylakoid membrane’ and ‘photosynthetic membrane’ pathways. Genes corresponding to molecular function were related to ‘DNA-binding transcription factor activity, RNA polymerase II-specific’ and ‘monooxygenase activity’ pathways (Fig. S3 A, B).

The KEGG pathway enrichment analysis was also carried out. The results demonstrated that the DEGs were mainly enriched in ‘photosynthesis’ and ‘flavonoid biosynthesis’ pathways in the M1/M2 pair, while in the ‘photosynthesis’ and ‘cutin, suberine and wax biosynthesis’ pathways in the M2/M3 pair (Fig. S3 C, D).

**Fig. 3:**
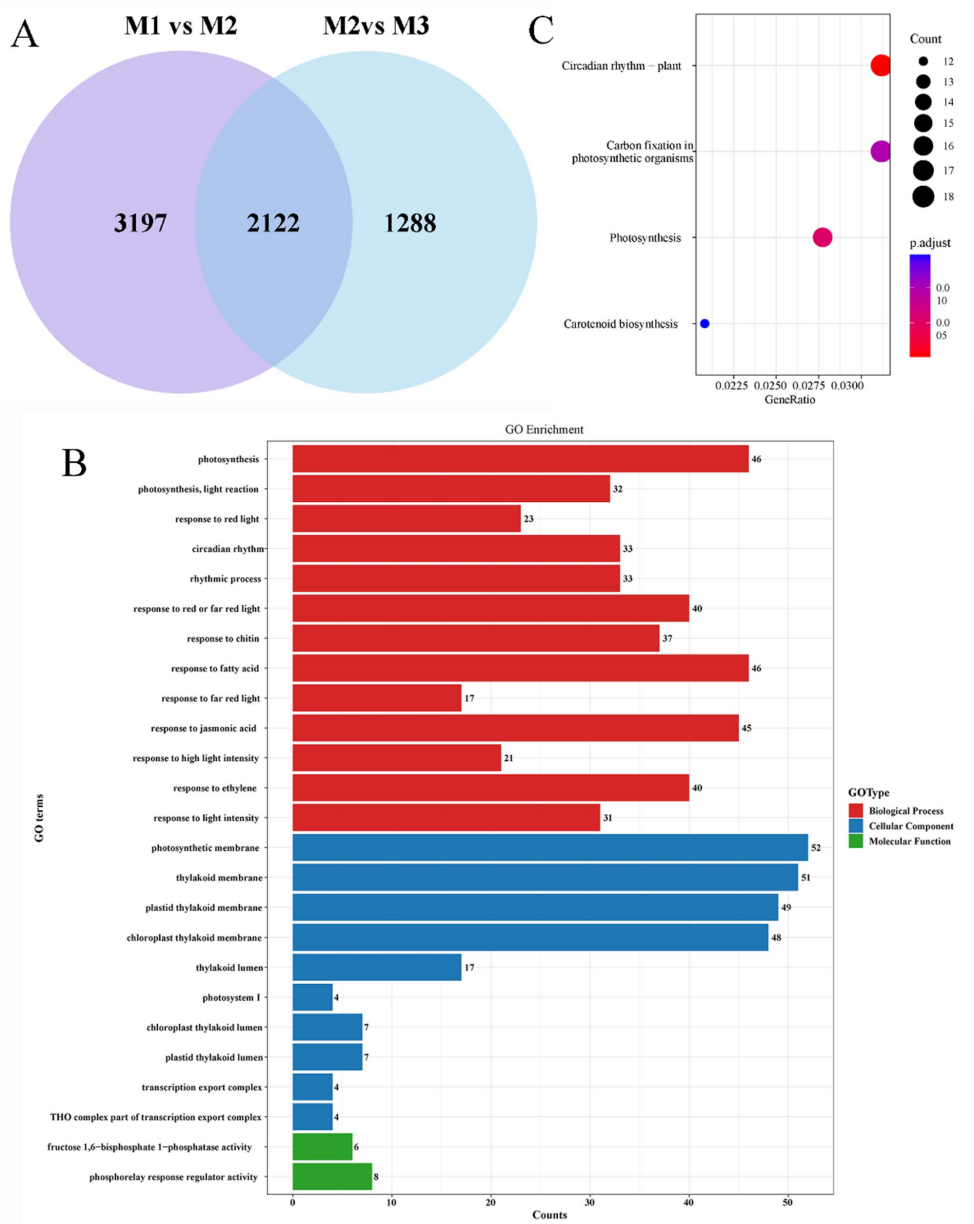
Differentially expressed genes (DEGs) and GO and KEGG analyses. **A**, Venn diagram indicating the number of DEGs in the M1/M2 and M2/M3 pairs. **B**, GO enrichment analysis of DEGs in the M1/M2 and M2/M3 pairs. **C**, KEGG pathway analysis of DEGs in the M1/M2 and M2/M3 pairs

Additionally, we investigated the co-expressed genes in the M1/M2 and M2/M3 pairs. A total of 2122 DEGs were co-expressed in both M1/M2 and M2/M2 pairs (Fig. 3A). GO analysis showed that these genes were mainly involved in biological processes and cellular component (Fig. 3B). For instance, most genes corresponding to biological processes were related to photosynthesis and responsive to light, suggesting that photosynthesis is involved in the development of ‘Fumei’ peels. Besides, some genes were responsive to plant hormones (such as jasmonic acid and ethylene). DEGs were enriched in the ‘response to the fatty acid’ pathway, indicating that the formation of cuticular wax on the ‘Fumei’ fruit is related to these genes (Fig. 3B). The KEGG pathway enrichment analysis showed that DEGs were mainly enriched in the ‘circadian rhythm’, ‘carbon fixation in photosynthetic organism’, ‘photosynthesis’ and ‘carotenoid biosynthesis’ pathways (Fig. 3C).

### DEGs related to the anthocyanin pathway in ‘fumei’ fruit

**Fig. 4:**
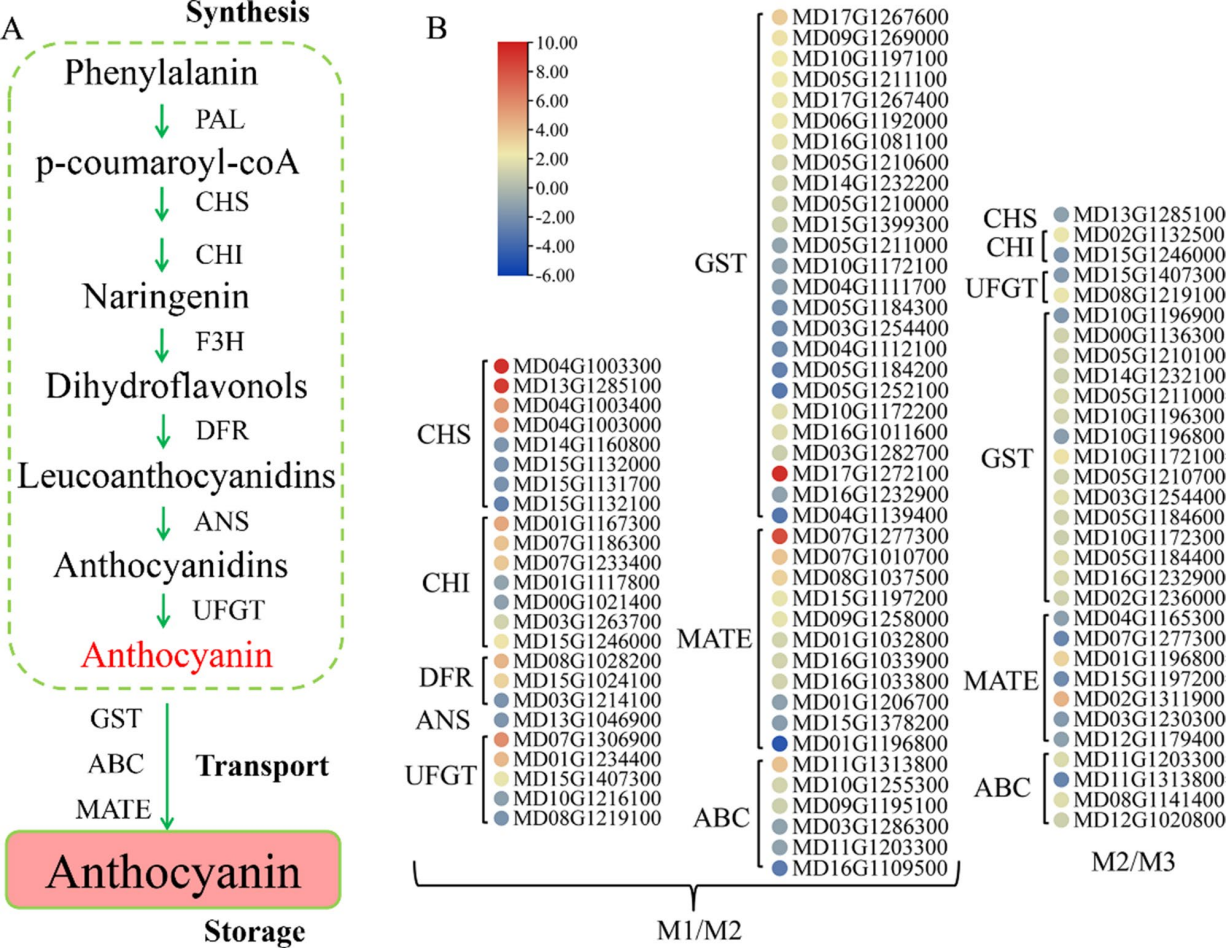
Expression patterns of genes related to biosynthesis and transport in the anthocyanin pathway in the M1/M2 and M2/M3 pairs. **A**, A model of anthocyanin biosynthetic pathway. **B**, Expression patterns of genes involved in the anthocyanin pathway in the M1/M2 and M2/M3 pairs. Value of log_2_ (fold change) was used in the heatmap. Red colour represents up-regulation, and blue colour represents down-regulation

To further investigate anthocyanin biosynthesis, DEGs related to biosynthesis and transport pathways were analyzed (Fig. 4A). As demonstrated, eight *CHS* genes, seven *CHI* genes, three *DFR* genes, one *ANS* gene and five *UFGT* genes were differentially expressed in the M1/M2 pair, while one *CHS* gene, two *CHI* genes and two *UFGT* genes were differentially expressed in the M2/M3 pair (Fig. 4B). Besides the structural genes, the transporter coding genes were also analyzed. The results suggested that 25 *GST* gens, 11 *MATE* genes and six *ABC* were differentially expressed in the M1/M2 pair, while 15 *GST* genes, seven *MATE* genes and four *ABC* genes were differentially expressed in the M2/M3 pair (Fig. 4B). In the M1/M2 pair, four *CHS* genes (MD04G1003300, MD13G1285100, MD04G1003400, MD04G1003000), three *CHI* genes (MD01G1167300, MD07G1186300, MD07G1233400), two *DFR* genes (MD08G1028200, MD15G1024100), two *UGFT* genes (MD07G1306900, MD01G1234400), one *GST* gene (MD17G1272100), one *MATE* gene (MD07G1277300) and one *ABC* gene (MD11G1313800) were highly expressed at Stage 3, indicating that the expression of these genes contributes to anthocyanin accumulation. In other words, the anthocyanin biosynthetic genes were up-regulated after bag removal.

### DEGs related to cuticular wax in ‘fumei’ fruit

**Fig. 5:**
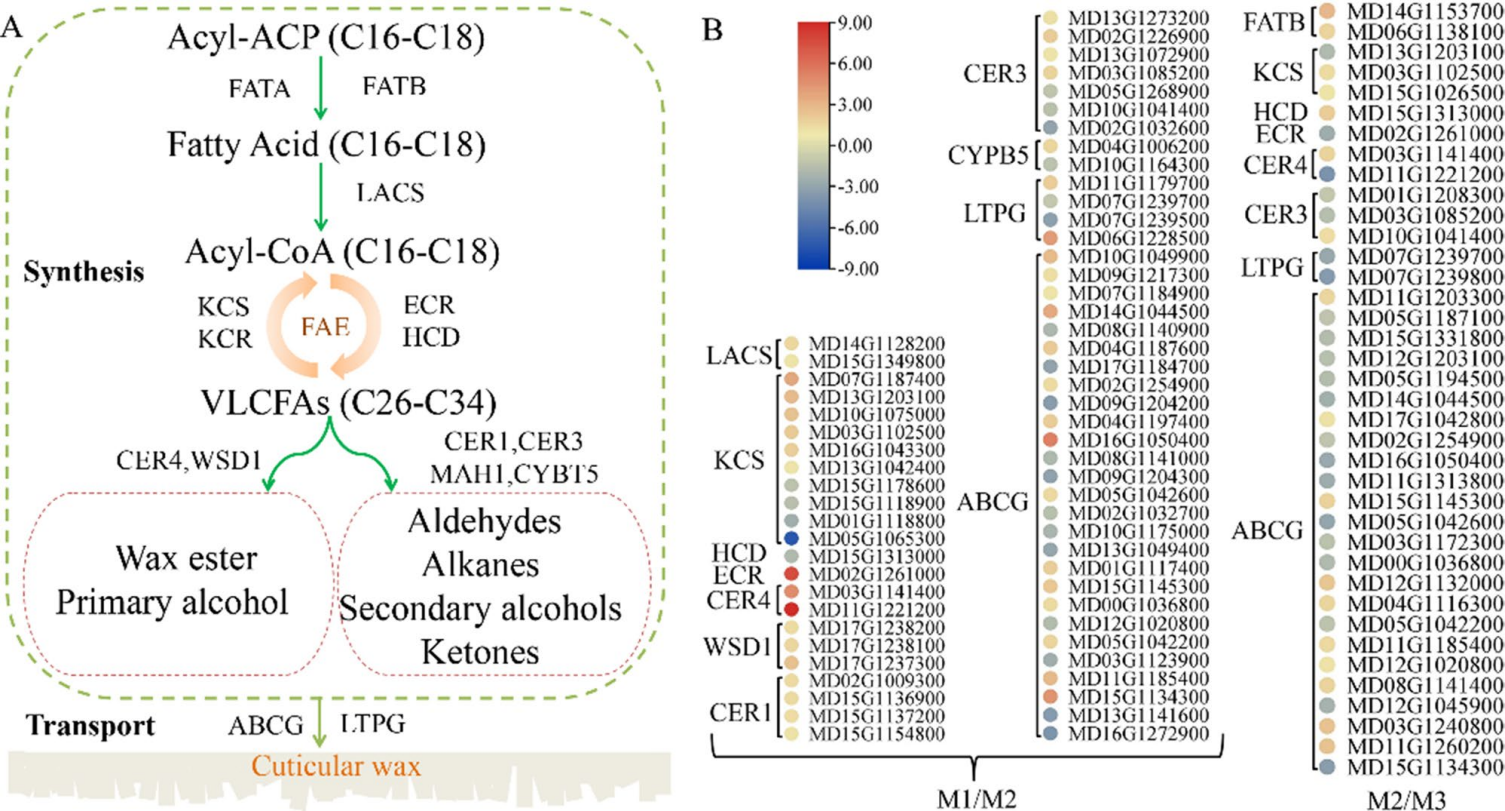
Expression patterns of genes related to cuticular wax biosynthesis in the M1/M2 and M2/M3 pairs. **A**, A model of cuticular wax biosynthetic pathway. **B**, Expression patterns of genes involved in the cuticular wax pathway in the M1/M2 and M2/M3 pairs. Value of log_2_ (fold change) was used in the heatmap. Red colour represents up-regulation, and blue colour represents down-regulation

DEGs involved in cuticular wax biosynthesis, including the synthesis and transport path, were also explored (Fig. 5A). The results showed that two *LACS* genes, ten *KCS* genes, one *HCD* gene, one *ECR* gene, two *CER4* genes, three *WSD1* genes, four *CER1* genes, seven *CER3* genes, and two *CYPB5* genes were differentially expressed in the M1/M2 pair, while two *FATB* genes, three *KCS* genes, one *HCD* gene, one *ECR* gene, two *CER4* genes, and three *CER3* genes were differentially expressed in the M2/M3 pair (Fig. 5B). Also, two transporters, including four *LTPG* genes and 27 *ABCG* genes, were differentially expressed in the M1/M2 pair, while two *LTPG* genes and 24 *ABCG* genes showed differential expression in the M2/M3 pair. Notably, one *ECR* gene (MD02G1261000) and two CER4 genes (MD03G1141400, MD11G1221200) were up-regulated at 12 DABR but down-regulated at 24 DBAR (Fig. 5B).

### Differentially expressed TFs involved in anthocyanin and cuticular wax accumulation

Anthocyanin biosynthesis is typically controlled by regulatory genes in plants. Hence, the regulatory genes involved in the anthocyanin biosynthetic pathway were investigated (Table 1). As indicated, *MYB1* (MD09G1278600), a key regulatory factor, was differentially expressed in both M1/M2 and M2/M3 pairs, and was up-regulated at 12 DABR and down-regulated at 24 DABR. Moreover, most MYB genes were differentially expressed in the M1/M2 pair and down-regulated in the development process. The *bHLH3* gene (MD11G1286900) exhibited a similar expression pattern to the *MYB1* gene, and interacted with MYB1 to generate a MBW complex in apple. *MYB9* was differentially expressed in the M2/M3 pair, indicating that MYB9 might regulate anthocyanin biosynthesis at the late stage of fruit development. Two MYB_SH[AL]QKY[RF] transcription factors, MdLUX (MD03G1086800) and MdPCL-like (MD11G1095900), exhibited opposite expression patterns and were down-regulated and then up-regulated (Table 1).

Regulatory genes involved in the cuticular wax biosynthetic pathway were also investigated (Table 2). As indicated, four MYB genes and one AP2 TF were differentially expressed. The *MYB16* gene (MD17G1051700) was differentially expressed in the M2/M3 pair, while the *MYB41* gene (MD09G1097700) was differentially expressed in the M1/M2 pair. *MYB94* (MD17G1050900), *MYB96* (MD14G1200100) and *WRI4* (MD17G1226700) exhibited consistent expression patterns, all of which were differentially expressed in both M1/M2 and M2/M3 pairs; moreover, they were up-regulated at 12 DABR and down-regulated at 0 DABR and 24 DABR. Notably, *MYB306-like* genes and *MYB94* genes belonged to the same ID (MD17G1050900), suggesting that they may simultaneously regulate the anthocyanin and cuticular wax biosynthesis in apples [32, 41].

**Table 1:** Transcription factors related DEGs that were involved in anthocyanin pathway

Name	ID	log_2_ foldchange in M1/M2	log_2_ foldchange in M2/M3
MYB1 [2]	MD09G1278600	4.82	-1.52
MYB3 [42]	MD15G1411200	2.00	-4.04
MYB9 [43]	MD08G1070700	nd	3.38
MYB11 [43]	MD09G1184000	2.14	nd
MYB90-like [44]	MD09G1278400	3.93	nd
MYB114 [35]	MD17G1261100	5.89	nd
MYB306-like [41]	MD17G1050900	1.15	-1.39
bHLH3 [45]	MD11G1286900	1.33	-1.14
LUX [46]	MD03G1086800	-2.87	2.43
PCL-like [46]	MD11G1095900	-1.89	1.59

Note: ‘nd’ means ‘not detected’ and representatives that the gene is not differently expressed in the comparison

**Table 2:** Transcription factors related DEGs that were involved in cuticular wax pathway

Name	ID	log_2_ foldchange in M1/M2	log_2_ foldchange in M2/M3
MYB16 [17]	MD17G1051700	nd	-1.31
MYB41 [47]	MD09G1097700	2.80	nd
MYB94 [32]	MD17G1050900	1.15	-1.39
MYB96 [48]	MD14G1200100	2.17	-1.39
WRI4 [49]	MD17G1226700	2.40	-1.50

Note: ‘nd’ means ‘not detected’ and representatives that the gene is not differently expressed in the comparison

### Identification of LncRNAs and DEGs in ‘fumei’ fruit

To identify all potential lncRNAs in ‘Fumei’ apple, 4080 lncRNAs were included in this study (Table S2). Compared with mRNAs, lncRNAs had shorter sequences, and most of them had sequence lengths less than 2500 bp (Fig. S3A). Moreover, most lncRNA transcripts had at least two exons, which were fewer than those of protein-coding mRNAs (Fig. S3B). Notably, the overall expression level of lncRNAs was lower than that of mRNAs (Fig. S3C).

**Fig. 6:**
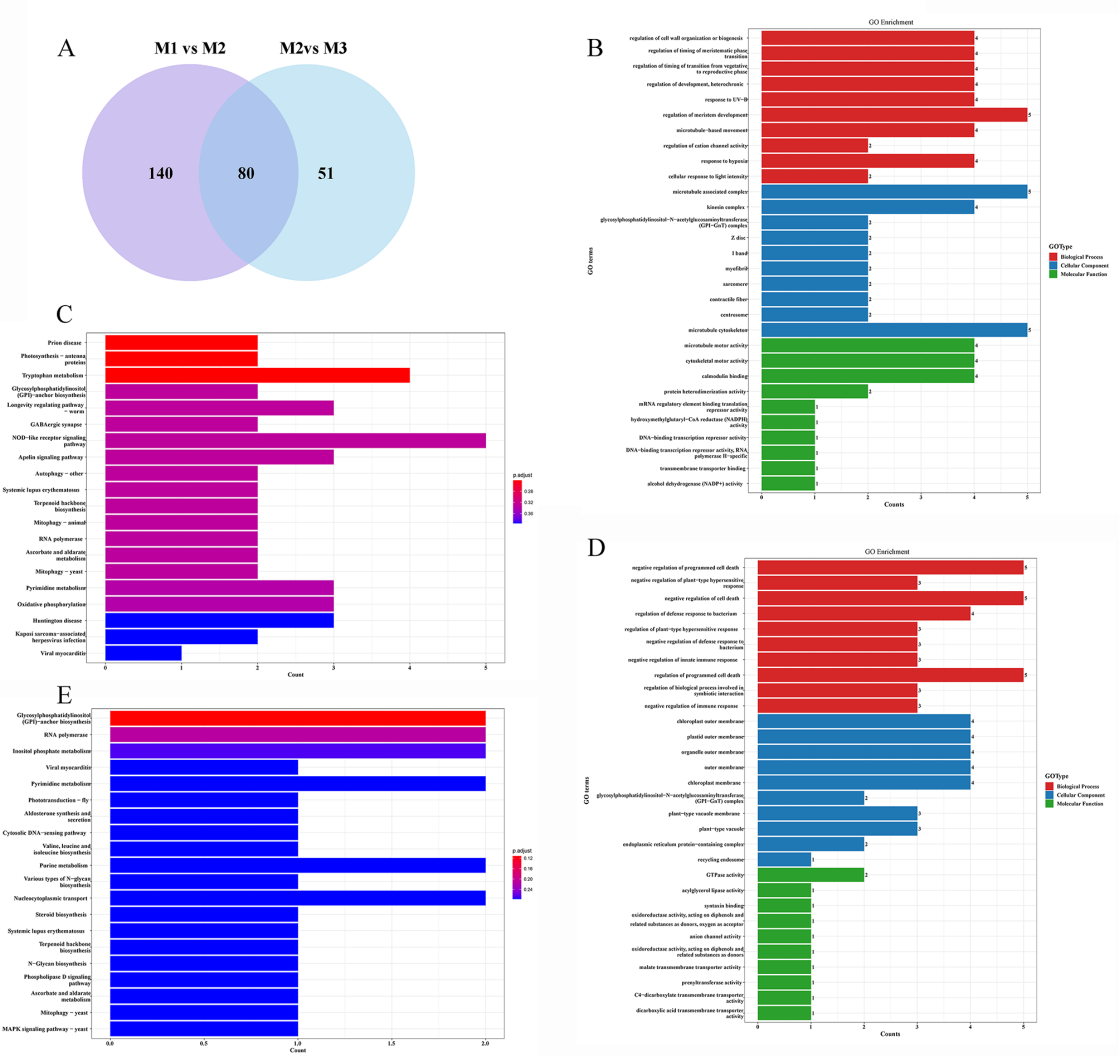
Numbers of DELs and GO and KEGG analyses of their target genes in the M1/M2 and M2/M3 pairs. **A**, Venn diagram indicating the number of DELs in the M1/M2 and M2/M3 pairs. **B**, GO enrichment analysis of target genes of DELs in the M1/M2 pair. C, KEGG pathway analysis of target genes of DELs in the M1/M2 pair. **D**, GO enrichment analysis of target genes of DELs in the M2/M3 pair. E, KEGG pathway analysis of target genes of DELs in the M2/M3 pair

According to the transcriptome data, 230 and 131 lncRNAs were differentially expressed in the M1/M2 and M2/M3 pairs, respectively (Fig. 6A). Specifically, 121 lncRNAs were up-regulated and 99 lncRNAs were down-regulated at 12 DABR compared with 0 DABR (Fig. S3A, C). In addition, 66 lncRNAs were up-regulated and 65 lncRNAs were down-regulated at 24 DABR compared with 12 DABR (Fig. S3B, C). Meanwhile, a total of 80 lncRNAs were differentially expressed in both pairs (Fig. 6A). It has been demonstrated that LncRNAs can regulate the expression of coding genes via *cis* and *trans* patterns. In this study, we also investigated the *cis* target genes. The results showed 178 and 80 lncRNA-targeted DEGs in the M1/M2 and M2/M3 pairs, respectively (Table S3 and S4). GO analysis showed that most of these target genes were involved in the ‘regulation of meristem development’, ‘response to UV-B’, ‘microtubule cytoskeleton’, and ‘calmodulin binding’ pathways in the M1/M2 pair (Fig. 6B). KEGG pathway enrichment analysis showed that these target genes were mainly enriched in the ‘Trypophan metabolism’, ‘NOD-like receptor signaling pathway’ and ‘Oxidative phosphorylation’ pathways (Fig. 6C). For the M2/M3 pair, GO analysis indicated that most target genes were involved in different processes related to cell death and extra-membrane component (Fig. 6D); KEGG pathway enrichment analysis showed that most target genes were enriched in the ‘RNA polymerase’, ‘Pyrimidine metabolism’, ‘Purine metabolism’ and ‘Nucleocytoplasmic transport’ pathways (Fig. 6E). Overall, most target genes were involved in amino acid synthesis and disease-resistant processes.

To clarify whether the DELs target the anthocyanin and cuticular wax biosynthetic genes, we investigated all targeted genes in the M1/M2 and M2/M3 pairs. The results showed that no genes were present in both anthocyanin and cuticular wax biosynthesis pathways. However, *MdMYB1* (MD09G1278600), a key regulatory gene in red apple, was targeted by the lncRNA *MSTRG.27523.1* (Table S3), indicating that *MSTRG.27523.1* may regulate anthocyanin biosynthesis via affecting *MdMYB1* in red apples. In addition, some target genes were involved in biotic and abiotic stress responses in apple. For instance, MdERF114 (MD16G1140800) interacts with MdMYB8 to improve resistance to *F. solani*, resulting in enhanced binding activity to the *MdPRX63* promoter in apple [50]. Two NLR coding genes (*MD02G1112400* and *MD02G1112800*) were also present in Table S3, suggesting that the biosynthesis of anthocyanin and cuticular wax at the late stage helps to improve plant resistance.

### Validation of gene expression

**Fig. 7:**
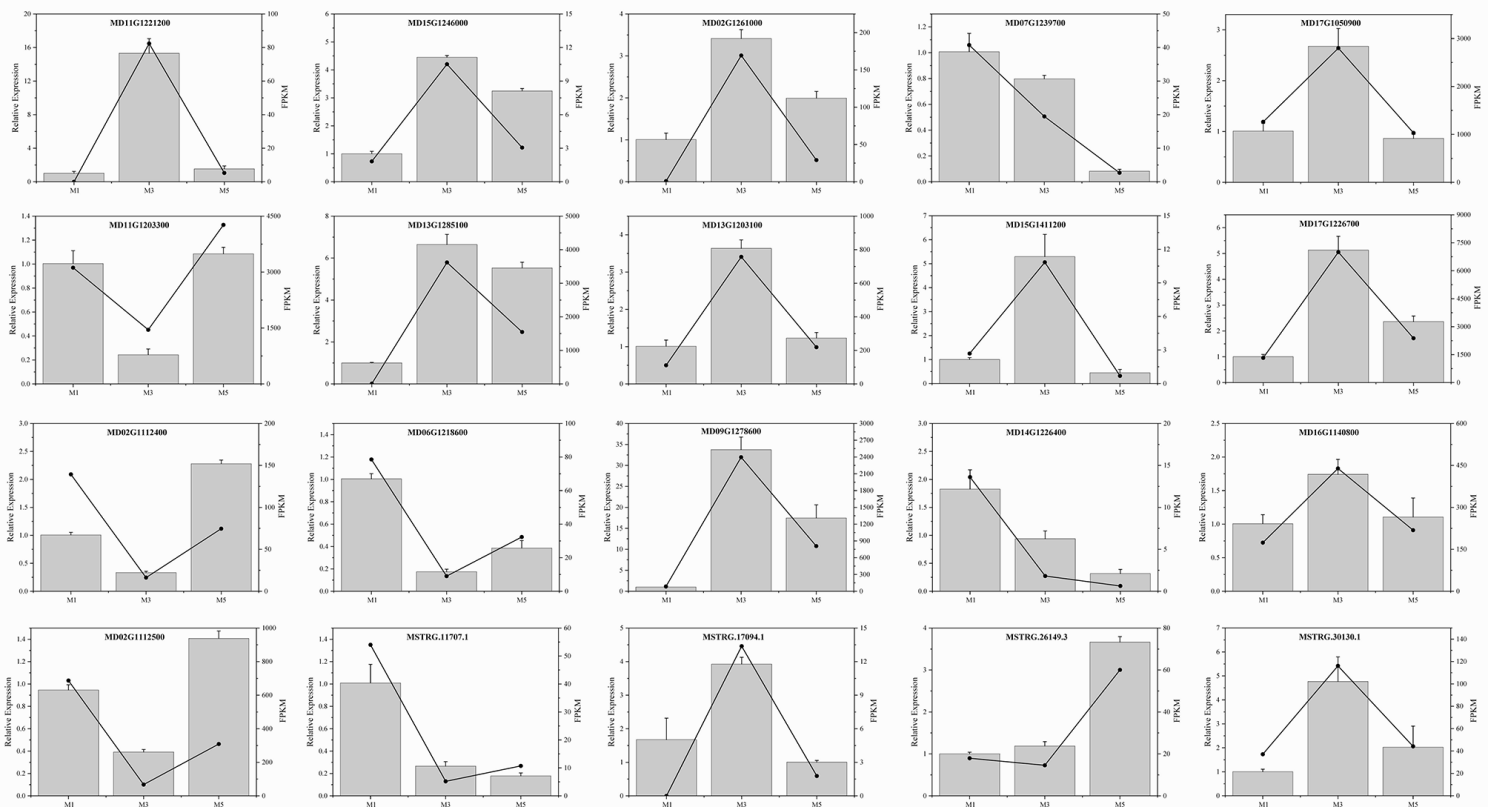
Validation of the expression of genes and their lncRNAs at different developmental stages. Error bar means ± SD, *n* ≥ 3

To validate the gene expression results obtained from the RNA-Seq, 15 mRNAs and 5 lncRNAs were selected for qRT-PCR verification. *MD02G1112400* and *MD02G1112500* were downregulated at 12 DABR and upregulated at 24 DABR, whereas their corresponding lncRNAs exhibited the opposite pattern. *MD09G1278600* and *MD16G1140800* showed expression patterns that closely matched those of their associated lncRNAs (Fig. 7). Despite some quantitative differences in expression levels, the trends of the expression levels of genes were similar in both the RNA-Seq and qRT-PCR data. These results confirm the reliability of the RNA-Seq data.

## Discussion

As a key fruit quality, the color of fruit significantly affects its economic value of apple. ‘Fumei’ is an apple variety with a fully-red phenotype and high wax content. In this study, we investigated the key regulator factors related to the high contents of anthocyanin and wax, and identified the lncRNA-mRNA regulator modules involved in these pathways.

Firstly, the contents of anthocyanin and cuticular wax in ‘Fumei’ were monitored during fruit development. Similar to most red apple varieties [2, 51], ‘Fumei’ showed high anthocyanin content at the late developmental stage (Fig. 1B). Anthocyanin is an important flavonoid determining the phenotype of red apples and can be readily detected. Indeed, high content of anthocyanin can enhance the appearance quality of fruits such as apple. Cuticular wax can improve the fruit glossiness and affect the sensory quality, conferring the fruit with higher economic value. ‘Fumei’ exhibited an attractive appearance due to their high anthocyanin and wax accumulation (Fig. S1). The composition of wax was different at different stages (Fig. 2B, C, D) due to its tissue or organ specificity. For instance, the leaves and stems of *Camelina sativa* have high contents of wax esters and triterpenoids/sterols, respectively, while its seed coats have high levels of primary alcohols [52]. In ‘Golden Delicious’ and ‘Red Delicious’ apples, alkane is the dominant wax component, which is consistent with our findings in ‘Fumei’ [53].

Anthocyanin is biosynthesized by structural genes on ER and transported to the vacuoles for storage. In this study, we analyzed the RNA-seq data at different developmental stages (0, 12, 24 DABR). The results showed that most DEGs were enriched in pathways related to ‘photosynthesis and response to light’ (Fig. S3A, B), indicating that light induces anthocyanin biosynthesis after bag removal. Previous studies have demonstrated that blue light can induce the expression of anthocyanin biosynthetic genes and anthocyanin accumulation in pear and strawberry [54, 55]. Moreover, in non-red apple cultivars, light-induced anthocyanin accumulation has been associated with epigenetic modifications, such as DNA methylation and histone modification [56, 57]. Overall, these findings collectively highlight the close relationship between light and anthocyanin biosynthesis in plants. To further investigate the regulatory mechanisms underlying anthocyanin biosynthesis, we identified seven transcription factors (TFs), including MYBs and bHLHs, that may play key regulatory roles in this pathway (Table 1). Unlike anthocyanins, cuticular wax was present from 0 DAFB, and its biosynthesis appears to be independent of light (Fig. 1C). However, previous studies have suggested that specific light wavelengths influence the accumulation and composition of cuticular wax in faba bean [58]. The cuticular wax in plants can prevent water loss and damages caused by biotic and abiotic stresses, including pathogens, pest, and UV radiation [59–61].

lncRNAs are ncRNAs with sequence lengths longer than 200 nt, and play key roles in flavonoid accumulation, fruit ripening, reproductive tissue development and responses to biotic and abiotic stresses in plants. For instance, *COLDWRAP* represses *FLC* expression by regulating H3K27m3 of the FLC promoter and develops an intragenic chromatin loop upstream of the *FLC* gene [62]. *MdLNC499* and *MdLNC610* were identified as a cis-regulator in light-induced anthocyanin accumulation [63, 64]. *BplncSIR1* affects salt resistance of *Betula platyphylla* by regulating *NpNAC2* to induce ROS scavenging and stomatal movement [65]. In this study, we analyzed DELs and their target genes at different developmental stages. A total of 230 and 131 DELs were identified in the M1/M2 and M2/M3 comparisons, respectively (Fig. 6A). Notably, MdMYB1, a key regulator of anthocyanin biosynthesis, was predicted to be regulated by MSTRG.27523.1 (Table S3). Additionally, a number of transcription factors were identified as putative targets of lncRNAs (Table S3); however, their specific roles in anthocyanin and cuticular wax biosynthesis remain unclear. Interestingly, no structural genes associated with either anthocyanin or cuticular wax biosynthetic pathways were identified among the predicted lncRNA targets. Overall, this study provides potential candidate lncRNAs and TFs that may regulate anthocyanin and cuticular wax biosynthesis during apple fruit development.

## Conclusions

In this study, we identified key lncRNAs and genes involved in apple coloration and wax formation through transcriptome analysis. The findings revealed that multiple protein-coding genes contribute to anthocyanin and wax biosynthesis, and that MdMYB1, a central regulator of anthocyanin biosynthesis, may be regulated by MSTRG.27523.1. Furthermore, we identified several potential lncRNA-mRNA regulatory interactions that may provide new insights into the molecular mechanisms governing anthocyanin and cuticular wax biosynthesis during apple fruit development.

## Electronic supplementary material

Below is the link to the electronic supplementary material.

Supplementary Material 1: Figure S1. Genealogy and phenotype of ‘Fumei’ apple

Supplementary Material 2: Figure S2. Numbers of DEGs identified in the M1/M2 and M2/M3 pairs. Volcano maps showing the upregulated and downregulated DEGs in the M1/M2 and M2/M3 pairs (A, B). Column charts showing numbers of upregulated and downregulated DEGs in the M1/M2 and M2/M3 pairs

Supplementary Material 3: Figure S3. GO enrichment and KEGG analysis of DEGs in the M1/M2 and M2/M3 pairs. A, GO enrichment analysis of DEGs in the M1/M2 pair. B, KEGG pathway analysis of DEGs in the M1/M2 pair. C, GO enrichment analysis of DEGs in the M2/M3 pair. D, KEGG pathway analysis of DEGs in the M2/M3 pair

Supplementary Material 4: Figure S4. Characteristics of lncRNAs identified in ‘Fumei’ apple. A, Length statistics of lncRNA and mRNA. B, Numbers of exons in lncRNAs and mRNAs. C, Average expression level of lncRNAs and mRNAs

Supplementary Material 5: Figure S5. Numbers of DELs identified in the M1/M2 and M2/M3 pairs. Volcano maps showing the upregulated and downregulated DELs in the M1/M2 and M2/M3 pairs (A, B). Column charts showing numbers of upregulated and downregulated DELs in the M1/M2 and M2/M3 pairs

Supplementary Material 6: Table S1. Primers used in this study. Table S2. IDs of lncRNAs identified in this study. Table S3. Details of lncRNA-targeted DEGs in M1/M2 pair. Table S4. Details of lncRNA-targeted DEGs in M2/M3 pair

## Data Availability

The raw sequencing data are publicly accessible under GSA No. CRA016451 at https://ngdc.cncb.ac.cn/gsa.

## References

[1] Zhang L, Hu J, Han X, Li J, Gao Y, Richards CM, Zhang C, Tian Y, Liu G, Gul H, et al. A high-quality Apple genome assembly reveals the association of a retrotransposon and red fruit colour. Nat Commun. 2019;10(1):1494. 30940818 10.1038/s41467-019-09518-x PMC6445120

[2] Jiang S, Wang N, Chen M, Zhang R, Sun Q, Xu H, Zhang Z, Wang Y, Sui X, Wang S, et al. Methylation of MdMYB1 locus mediated by RdDM pathway regulates anthocyanin biosynthesis in Apple. Plant Biotechnol J. 2020;18(8):1736–48. 31930634 10.1111/pbi.13337 PMC7336386

[3] Ban Y, Honda C, Hatsuyama Y, Igarashi M, Bessho H, Moriguchi T. Isolation and functional analysis of a MYB transcription factor gene that is a key regulator for the development of red coloration in Apple skin. Plant Cell Physiol. 2007;48(7):958–70. 17526919 10.1093/pcp/pcm066

[4] Wang Z, Liu S, Huo W, Chen M, Zhang Y, Jiang S. Transcriptome and metabolome analyses reveal phenotype formation differences between russet and non-russet apples. Front Plant Sci. 2022;13:1057226. 36426145 10.3389/fpls.2022.1057226 PMC9678910

[5] Jiang S, Chen M, Wang Z, Ren Y, Wang B, Zhu J, Zhang Y. Advances in Understanding the causes, molecular mechanism, and perspectives of russeting on tree fruit. Front Plant Sci 2022, 13:834109. 10.3389/fpls.2022.834109 PMC8919063 35295640

[6] Xu W, Dubos C, Lepiniec L. Transcriptional control of flavonoid biosynthesis by MYB-bHLH-WDR complexes. Trends Plant Sci. 2015;20(3):176–85. 25577424 10.1016/j.tplants.2014.12.001

[7] Gomez C, Terrier N, Torregrosa L, Vialet S, Fournier-Level A, Verries C, Souquet JM, Mazauric JP, Klein M, Cheynier V, et al. Grapevine MATE-type proteins act as vacuolar H+-dependent acylated anthocyanin transporters. Plant Physiol. 2009;150(1):402–15. 19297587 10.1104/pp.109.135624 PMC2675721

[8] Sun Y, Li H, Huang J-R. Arabidopsis TT19 functions as a carrier to transport anthocyanin from the cytosol to tonoplasts. Mol Plant. 2012;5(2):387–400. 22201047 10.1093/mp/ssr110

[9] Francisco RM, Regalado A, Ageorges A, Burla BJ, Bassin B, Eisenach C, Zarrouk O, Vialet S, Marlin T, Chaves MM, et al. ABCC1, an ATP binding cassette protein from grape Berry, transports Anthocyanidin 3-O-Glucosides. Plant Cell. 2013;25(5):1840–54. 23723325 10.1105/tpc.112.102152 PMC3694709

[10] Baudry A, Heim MA, Dubreucq B, Caboche M, Weisshaar B, Lepiniec L. TT2, TT8, and TTG1 synergistically specify the expression of BANYULS and Proanthocyanidin biosynthesis in Arabidopsis thaliana. Plant J. 2004;39(3):366–80. 15255866 10.1111/j.1365-313X.2004.02138.x

[11] Marinova K, Pourcel L, Weder B, Schwarz M, Barron D, Routaboul JM, Debeaujon I, Klein M. The Arabidopsis MATE transporter TT12 acts as a vacuolar flavonoid/h++ -antiporter active in proanthocyanidin-accumulating cells of the seed coat. Plant Cell. 2007;19(6):2023–38. 17601828 10.1105/tpc.106.046029 PMC1955721

[12] Jiang S, Chen M, He N, Chen X, Wang N, Sun Q, Zhang T, Xu H, Fang H, Wang Y, et al. MdGSTF6, activated by MdMYB1, plays an essential role in anthocyanin accumulation in Apple. Hortic Res. 2019;6:40. 30854214 10.1038/s41438-019-0118-6 PMC6395711

[13] Baudry A, Heim MA, Dubreucq B, Caboche M, Weisshaar B, Lepiniec LJPJ. TT2, TT8, and TTG1 synergistically specify the expression of BANYULS and Proanthocyanidin biosynthesis in Arabidopsis thaliana. Plant J. 2004, 39(3):366–80. 10.1111/j.1365-313X.2004.02138.x 15255866

[14] Takos AM, Jaffe FW, Jacob SR, Bogs J, Robinson SP, Walker AR. Light-induced expression of a MYB gene regulates anthocyanin biosynthesis in red apples. Plant Physiol. 2006;142(3):1216–32. 17012405 10.1104/pp.106.088104 PMC1630764

[15] Espley RV, Hellens RP, Putterill J, Stevenson DE, Kutty-Amma S, Allan AC. Red colouration in Apple fruit is due to the activity of the MYB transcription factor, MdMYB10. Plant J. 2007;49(3):414–27. 17181777 10.1111/j.1365-313X.2006.02964.x PMC1865000

[16] Xu H, Zou Q, Yang G, Jiang S, Chen X. MdMYB6 regulates anthocyanin formation in Apple both through direct Inhibition of the biosynthesis pathway and through substrate removal. 园艺研究(英文). 2020;7(1):17. 32377362 10.1038/s41438-020-0294-4 PMC7195469

[17] Hu Y, Cheng H, Zhang Y, Zhang J, Niu S, Wang X, Li W, Zhang J, Yao Y. The MdMYB16/MdMYB1-miR7125-MdCCR module regulates the homeostasis between anthocyanin and lignin biosynthesis during light induction in Apple. New Phytol. 2021;231(3):1105–22. 33908060 10.1111/nph.17431

[18] Paolocci F, Robbins MP, Passeri V, Hauck B, Morris P, Rubini A, Arcioni S, Damiani F. The strawberry transcription factor FaMYB1 inhibits the biosynthesis of proanthocyanidins in Lotus corniculatus leaves. J Exp Bot. 2011, 62(3):1189–2100. 10.1093/jxb/erq344 21041370

[19] Xu F, Ning Y, Zhang W, Liao Y, Li L, Cheng H, Cheng S. An R2R3-MYB transcription factor as a negative regulator of the flavonoid biosynthesis pathway in Ginkgo biloba. Funct Integr Genom. 2014;14(1):177–89. 10.1007/s10142-013-0352-1 24306138

[20] Bai S, Tao R, Tang Y, Yin L, Ma Y, Ni J, Yan X, Yang Q, Wu Z, Zeng Y, et al. BBX16, a B-box protein, positively regulates light-induced anthocyanin accumulation by activating MYB10 in red Pear. Plant Biotechnol J. 2019;17(10):1985–97. 30963689 10.1111/pbi.13114 PMC6737026

[21] An JP, Wang XF, Li YY, Song LQ, Zhao LL, You CX, Hao YJ. EIN3-LIKE1, MYB1, and ETHYLENE RESPONSE FACTOR3 act in a regulatory loop that synergistically modulates ethylene biosynthesis and anthocyanin accumulation. Plant Physiol. 2018;178(2):808–23. 29925585 10.1104/pp.18.00068 PMC6181056

[22] Brown AP, Affleck V, Slabas FAR. Tandem affinity purification tagging of fatty acid biosynthetic enzymes in Synechocystis Sp. PCC6803 and Arabidopsis thaliana. J Exp Bot. 2006;57(7):1563–71. 16551681 10.1093/jxb/erj150

[23] Kunst L, Samuels L. Plant cuticles Shine: advances in wax biosynthesis and export. Curr Opin Plant Biol. 2009;12(6):721–7. 19864175 10.1016/j.pbi.2009.09.009

[24] Libeisson Y, Shorrosh B, Beisson F, Andersson MX, Arondel V, Bates PD, Baud S, Bird D, Debono A, Durrett TP. BioOne Online Journals - Acyl-Lipid Metabolism. *Bioone*.

[25] Bernard A, Domergue F, Pascal S, Jetter R, Renne C, Faure JD, Haslam RP, Napier JA, Lessire R, Joubes J. Reconstitution of plant alkane biosynthesis in yeast demonstrates that Arabidopsis ECERIFERUM1 and ECERIFERUM3 are core components of a Very-Long-Chain alkane synthesis complex. PLANT CELL ONLINE. 2012;24(7):3106–18. 10.1105/tpc.112.099796 PMC3426135 22773744

[26] Bonaventure G, Salas, Joaquin J, Pollard, Michael R, Ohlrogge J. Disruption of the FATB gene in Arabidopsis demonstrates an essential role of saturated fatty acids in plant growth. Plant Cell. 2003;15(4):1020–1020. 12671095 10.1105/tpc.008946 PMC152346

[27] McFarlane HE, Watanabe Y, Yang W, Huang Y. J.: Golgi- and Trans-Golgi Network-Mediated vesicle trafficking is required for wax secretion from epidermal cells. PLANT PHYSIOLOGY; 2014. 10.1104/pp.113.234583 PMC3938617 24468625

[28] Kim H. Characterization of glycosylphosphatidylinositol-anchored lipid transfer protein 2 (LTPG2) and overlapping function between LTPG/LTPG1 and LTPG2 in cuticular wax export or accumulation in Arabidopsis thaliana. Plant Cell Physiol. 2012;53(8):1391. 22891199 10.1093/pcp/pcs083

[29] Aharoni A, Dixit S, Jetter R, Thoenes E, Arkel GV, Pereira A. The shine clade of AP2 domain transcription factors activates wax biosynthesis, alters cuticle properties, and confers drought tolerance when overexpressed in Arabidopsis. Plant Cell. 2004, 16(9):2463–2680. 10.1105/tpc.104.022897 PMC520946 15319479

[30] Broun, Pierre, Poindexter, Patricia, Osborne, Erin, Jiang. Cai-Zhong, Riechmann, Luis J: WIN1, a transcriptional activator of epidermal wax accumulation in Arabidopsis. 10.1073/pnas.0305574101 PMC384811 15070782

[31] Seo PJ, Lee SB, Suh MC, Park MJ, Go YS, Park CM. The MYB96 transcription factor regulates cuticular wax biosynthesis under drought conditions in Arabidopsis. Plant Cell. 2011;23(7):1138–52. 21398568 10.1105/tpc.111.083485 PMC3082259

[32] Jiang L, Zhang D, Liu C, Shen W, He J, Yue Q, Niu C, Yang F, Li X, Shen X, et al. MdGH3.6 is targeted by MdMYB94 and plays a negative role in Apple water-deficit stress tolerance. Plant J. 2022;109(5):1271–89. 34918398 10.1111/tpj.15631

[33] Zhang M, Wang J, Liu R, Liu H, Yang H, Zhu Z, Xu R, Wang P, Deng X, Xue S, Zhu F, Cheng Y. CsMYB96 confers resistance to water loss in citrus fruit by simultaneous regulation of water transport and wax biosynthesis. J Exp Bot. 2022, 73(3):953–966. 10.1093/jxb/erab420 34599807

[34] Jiang H, Qi CH, Gao HN, Feng ZQ, Wu YT, Xu XX, Cui JY, Wang XF, Lv YH, Gao WS, et al. MdBT2 regulates nitrogen-mediated cuticular wax biosynthesis via a MdMYB106-MdCER2L1 signalling pathway in Apple. Nat Plants. 2024;10(1):131–44. 38172573 10.1038/s41477-023-01587-7

[35] Jiang S, Sun Q, Zhang T, Liu W, Wang N, Chen X. MdMYB114 regulates anthocyanin biosynthesis and functions downstream of MdbZIP4-like in Apple fruit. J Plant Physiol. 2021;257:153353. 33352460 10.1016/j.jplph.2020.153353

[36] Daccord N, Celton J-M, Linsmith G, Becker C, Choisne N, Schijlen E, van de Geest H, Bianco L, Micheletti D, Velasco R, et al. High-quality de Novo assembly of the Apple genome and methylome dynamics of early fruit development. Nat Genet. 2017;49(7):1099–106. 28581499 10.1038/ng.3886

[37] Kim D, Langmead B, Salzberg SL. HISAT: a fast spliced aligner with low memory requirements. Nat Methods. 2015, 12(4):357–360. 10.1038/nmeth.3317 PMC4655817 25751142

[38] Kong L, Zhang Y, Ye ZQ, Liu XQ, Zhao SQ, Wei L, Gao G. CPC: assess the protein-coding potential of transcripts using sequence features and support vector machine. Nucleic Acids Res. 2007, 35:345–349 10.1093/nar/gkm391 PMC1933232 17631615

[39] Love MI, Huber W, Anders S. Moderated Estimation of fold change and dispersion for RNA-seq data with DESeq2. Genome Biol. 2014;15(12):550. 25516281 10.1186/s13059-014-0550-8 PMC4302049

[40] Livak KJ, Schmittgen TD. Analysis of relative gene expression data using real-time quantitative PCR and the 2(-Delta Delta C(T)) method. Methods. 2001;25(4):402–8. 11846609 10.1006/meth.2001.1262

[41] Wang S, Zhang Z, Li LX, Wang HB, Zhou H, Chen XS, Feng SQ. Apple MdMYB306-like inhibits anthocyanin synthesis by directly interacting with MdMYB17 and MdbHLH33. Plant J. 2022;110(4):1021–34. 35220614 10.1111/tpj.15720

[42] Vimolmangkang S, Han Y, Wei G, Korban SS. An Apple MYB transcription factor, MdMYB3, is involved in regulation of anthocyanin biosynthesis and flower development. BMC Plant Biol 2013, 13(1). 10.1186/1471-2229-13-176 PMC3833268 24199943

[43] An XH, Tian Y, Chen KQ, Liu XJ, Liu DD, Xie XB, Cheng CG, Cong PH, Hao YJ. MdMYB9 and MdMYB11 are involved in the regulation of the JA-induced biosynthesis of anthocyanin and Proanthocyanidin in apples. Plant Cell Physiol. 2015;56(4):650–62. 25527830 10.1093/pcp/pcu205

[44] Sun C, Wang C, Zhang W, Liu S, Wang W, Yu X, Song T, Yu M, Yu W, Qu S. The R2R3-type MYB transcription factor MdMYB90-like is responsible for the enhanced skin color of an apple bud sport mutant. Hortic Res. 2021, 8(1):156. 10.1038/s41438-021-00590-3 PMC8245648 34193856

[45] Xie XB, Li S, Zhang RF, Zhao J, Chen YC, Zhao Q, Yao YX, You CX, Zhang XS, Hao YJ. The bHLH transcription factor MdbHLH3 promotes anthocyanin accumulation and fruit colouration in response to low temperature in apples. Plant Cell Environ. 2012;35(11):1884–97. 22519753 10.1111/j.1365-3040.2012.02523.x

[46] Li WF, Ning GX, Zuo CW, Chu MY, Yang SJ, Ma ZH, Zhou Q, Mao J, Chen BH. MYB_SH[AL]QKY[RF] transcription factors mdlux and MdPCL-like promote anthocyanin accumulation through DNA hypomethylation and MdF3H activation in Apple. Tree Physiol. 2021;41(5):836–48. 33171489 10.1093/treephys/tpaa156

[47] Kosma DK, Murmu J, Razeq FM, Santos P, Bourgault R, Molina I, Rowland O. AtMYB41 activates ectopic Suberin synthesis and assembly in multiple plant species and cell types. Plant J. 2014;80(2):216–29. 25060192 10.1111/tpj.12624 PMC4321041

[48] Zhang M, Wang J, Liu R, Liu H, Yang H, Zhu Z, Xu R, Wang P, Deng X, Xue S, et al. CsMYB96 confers resistance to water loss in citrus fruit by simultaneous regulation of water transport and wax biosynthesis. J Exp Bot. 2022;73(3):953–66. 34599807 10.1093/jxb/erab420

[49] Park CS, Go YS, Suh MC. Cuticular wax biosynthesis is positively regulated by WRINKLED4, an AP2/ERF-type transcription factor, in Arabidopsis stems. Plant J. 2016,88(2):257–270 10.1111/tpj.13248 27337244

[50] Liu Y, Liu Q, Li X, Zhang Z, Ai S, Liu C, Ma F, Li C. MdERF114 enhances the resistance of Apple roots to fusarium Solani by regulating the transcription of MdPRX63. Plant Physiol. 2023;192(3):2015–29. 36721923 10.1093/plphys/kiad057 PMC10315273

[51] Sun Q, Jiang S, Zhang T, Xu H, Fang H, Zhang J, Su M, Wang Y, Zhang Z, Wang N, et al. Apple NAC transcription factor MdNAC52 regulates biosynthesis of anthocyanin and Proanthocyanidin through MdMYB9 and MdMYB11. Plant Sci. 2019;289:110286. 31623786 10.1016/j.plantsci.2019.110286

[52] Razeq FM, Kosma DK, Rowland O, Molina I. Extracellular lipids of Camelina sativa: characterization of chloroform-extractable waxes from aerial and subterranean surfaces. Phytochemistry. 2014;106:188–96. 25081105 10.1016/j.phytochem.2014.06.018

[53] Wang YX, Wang XJ, Cao Y, Zhong MS, Zhang J, Yu K, Li ZW, You CX, Li YY. Chemical composition and morphology of Apple cuticular wax during fruit growth and development. 果树研究(英文). 2022;2(1):41–51.37676543

[54] Tao R, Bai S, Ni J, Yang Q, Zhao Y, Teng Y. The blue light signal transduction pathway is involved in anthocyanin accumulation in ‘red Zaosu’ Pear. Planta. 2018;248(1):37–48. 29546452 10.1007/s00425-018-2877-y

[55] Zhang Y, Jiang L, Li Y, Chen Q, Ye Y, Zhang Y, Luo Y, Sun B, Wang X, Tang H. Effect of red and blue light on anthocyanin accumulation and differential gene expression in strawberry (Fragaria × ananassa). Molecules 2018, 23(4). 10.3390/molecules23040820 PMC6017741 29614032

[56] Bai S, Tuan PA, Saito T, Honda C, Hatsuyama Y, Ito A, Moriguchi T. Epigenetic regulation of MdMYB1 is associated with paper bagging-induced red pigmentation of apples. Planta. 2016;244(3):573–86. 27105885 10.1007/s00425-016-2524-4

[57] Ma C, Jing C, Chang B, Yan J, Liang B, Liu L, Yang Y, Zhao Z. The effect of promoter methylation on MdMYB1 expression determines the level of anthocyanin accumulation in skins of two non-red Apple cultivars. BMC Plant Biol. 2018;18(1):108. 29871614 10.1186/s12870-018-1320-7 PMC5989451

[58] Huang L, Xiao Q, Zhao X, Wang D, Wei L, Li X, Liu Y, He Z, Kang L, Guo Y. Responses of cuticular waxes of faba bean to light wavelengths and selection of candidate genes for cuticular wax biosynthesis. Plant Genome. 2020;13(3):e20058. 33124766 10.1002/tpg2.20058 PMC12807296

[59] Fukuda S, Satoh A, Kasahara H, Matsuyama H, Takeuchi Y. Effects of ultraviolet-B irradiation on the cuticular wax of cucumber (Cucumis sativus) cotyledons. J Plant Res. 2008;121(2):179–89. 18217194 10.1007/s10265-007-0143-7

[60] Hansjakob A, Bischof S, Bringmann G, Hildebrandt RU. Very-long-chain aldehydes promote in vitro Prepenetration processes of Blumeria Graminis in a dose- and chain length-dependent manner. New Phytol. 2010;188(4):1039–54. 20731784 10.1111/j.1469-8137.2010.03419.x

[61] Borodich FM, Gorb EV, SN. Fracture behaviour of plant epicuticular wax crystals and its role in preventing insect attachment:a theoretical approach. APPL PHYS A-MATER. 2010;2010(1001–):63–71.

[62] Kim DH, Sung S. Coordination of the vernalization response through a VIN3 and FLC gene family regulatory network in Arabidopsis. Plant Cell. 2013;25(2):454–69. 23417034 10.1105/tpc.112.104760 PMC3608771

[63] Ma H, Yang T, Li Y, Zhang J, Wu T, Song T, Yao Y, Tian J. The long noncoding RNA MdLNC499 bridges MdWRKY1 and MdERF109 function to regulate early-stage light-induced anthocyanin accumulation in Apple fruit. Plant Cell. 2021;33(10):3309–30. 34270784 10.1093/plcell/koab188 PMC8505877

[64] Yu J, Qiu K, Sun W, Yang T, Wu T, Song T, Zhang J, Yao Y, Tian J. A long noncoding RNA functions in high-light-induced anthocyanin accumulation in Apple by activating ethylene synthesis. Plant Physiol. 2022;189(1):66–83. 35148400 10.1093/plphys/kiac049 PMC9070812

[65] Jia Y, Zhao H, Niu Y, Wang Y. Long noncoding RNA from Betula platyphylla, BplncSIR1, confers salt tolerance by regulating BpNAC2 to mediate reactive oxygen species scavenging and stomatal movement. Plant Biotechnol J. 2024;22(1):48–65. 37697445 10.1111/pbi.14164 PMC10754008

