# Supplementary figures and images for: Combined analysis of lncRNAs and mRNAs associated with coloration and wax formation during ‘Fumei’ Apple development
Source: BMC Plant Biol. 2025 Apr 21;25:498. doi: 10.1186/s12870-025-06545-3 (PMC12010529; doi:10.1186/s12870-025-06545-3)

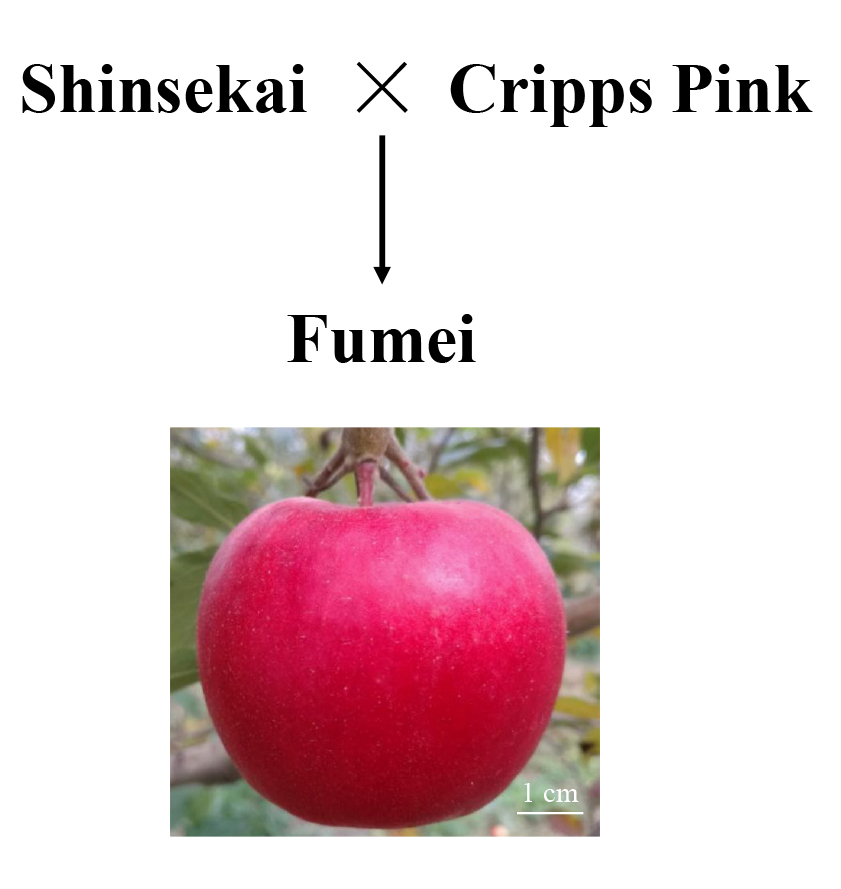

Supplement: Supplementary file 1 — Supplementary Material 1: Figure S1. Genealogy and phenotype of ‘Fumei’ apple [file 12870_2025_6545_MOESM1_ESM.jpg]

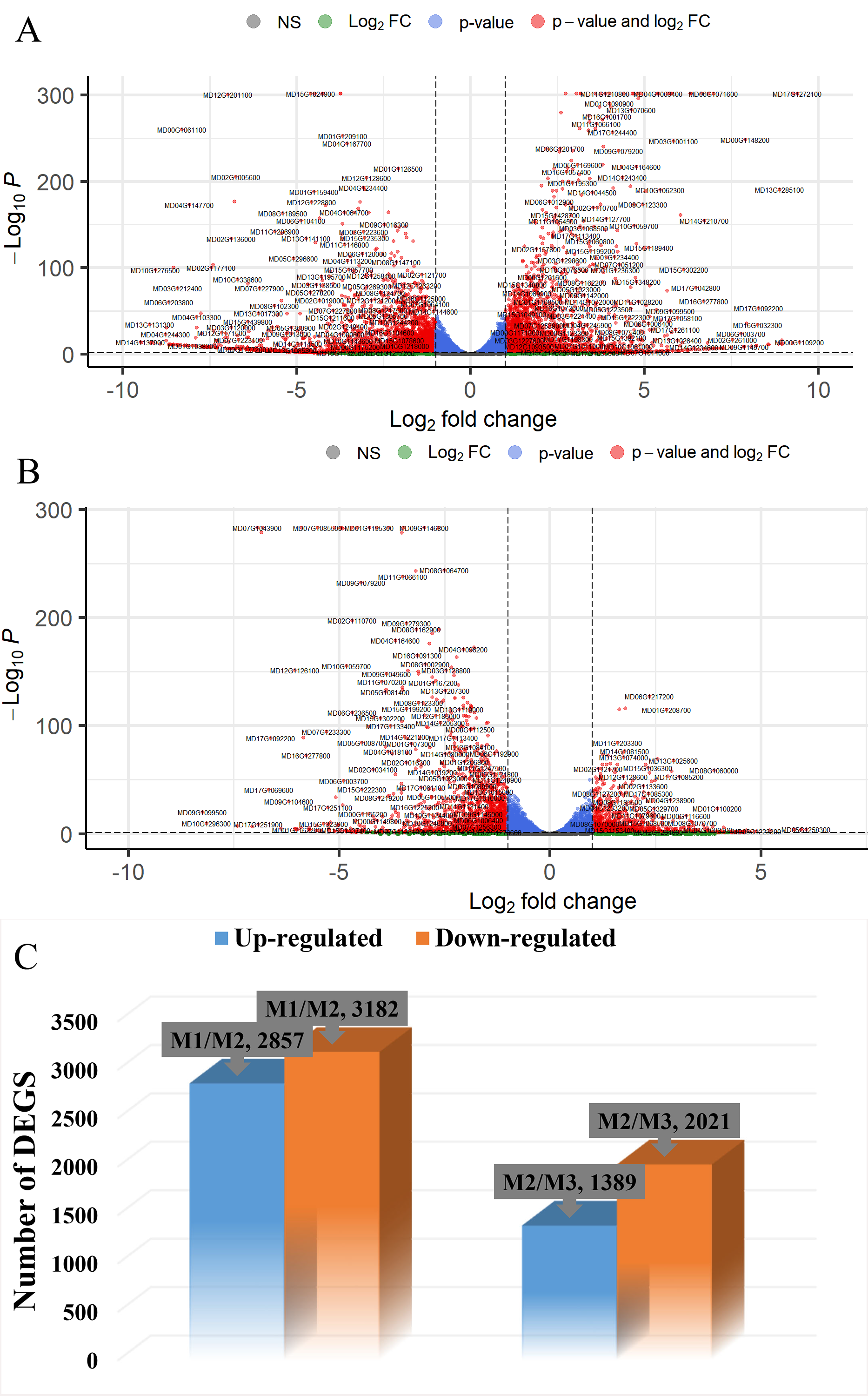

Supplement: Supplementary file 2 — Supplementary Material 2: Figure S2. Numbers of DEGs identified in the M1/M2 and M2/M3 pairs. Volcano maps showing the upregulated and downregulated DEGs in the M1/M2 and M2/M3 pairs (A, B). Column charts showing numbers of upregulated and downregulated DEGs in the M1/M2 and M2/M3 pairs [file 12870_2025_6545_MOESM2_ESM.tif]

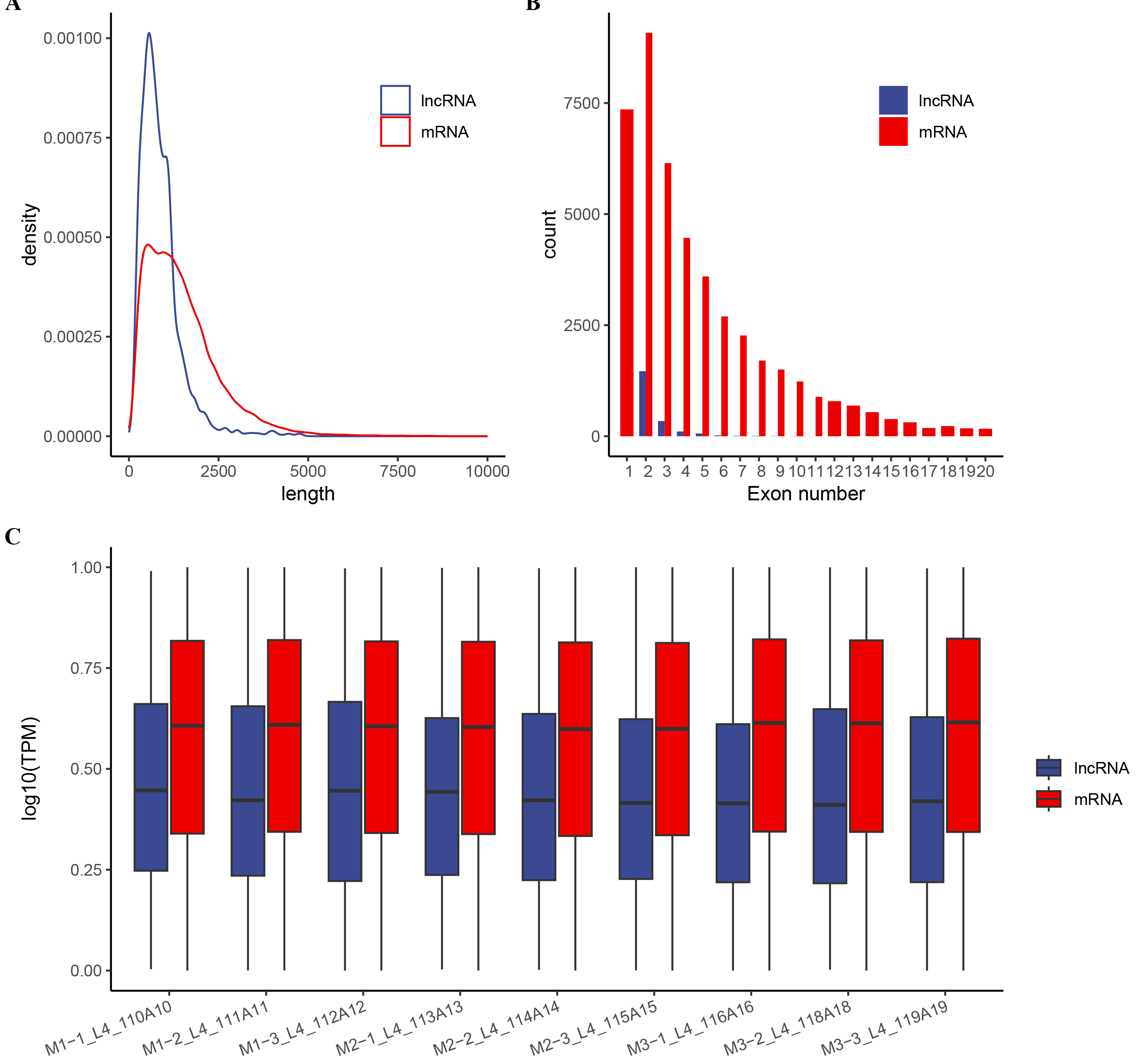

Supplement: Supplementary file 3 — Supplementary Material 3: Figure S3. GO enrichment and KEGG analysis of DEGs in the M1/M2 and M2/M3 pairs. A, GO enrichment analysis of DEGs in the M1/M2 pair. B, KEGG pathway analysis of DEGs in the M1/M2 pair. C, GO enrichment analysis of DEGs in the M2/M3 pair. D, KEGG pathway analysis of DEGs in the M2/M3 pair [file 12870_2025_6545_MOESM3_ESM.tif]

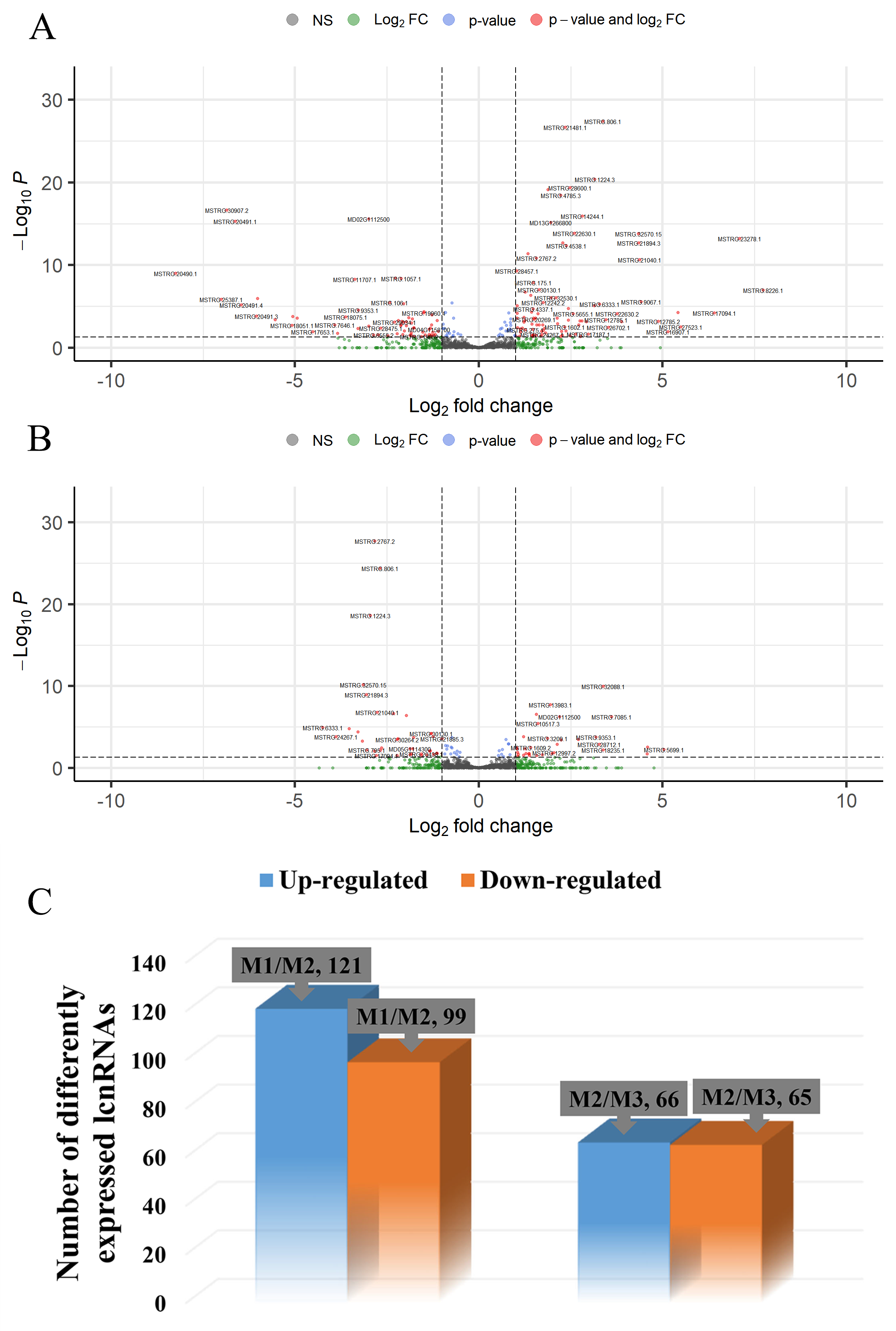

Supplement: Supplementary file 4 — Supplementary Material 4: Figure S4. Characteristics of lncRNAs identified in ‘Fumei’ apple. A, Length statistics of lncRNA and mRNA. B, Numbers of exons in lncRNAs and mRNAs. C, Average expression level of lncRNAs and mRNAs [file 12870_2025_6545_MOESM4_ESM.tif]
